# Comparing Population-General and Sport-Specific Correlates of Disordered Eating Amongst Elite Athletes: A Cross-Sectional Study

**DOI:** 10.1186/s40798-024-00791-9

**Published:** 2024-11-12

**Authors:** Scott J. Fatt, Emma George, Phillipa Hay, Nikki Jeacocke, Deborah Mitchison

**Affiliations:** 1 Translational Health Research Institute, School of Medicine, Western Sydney University, Sydney, Australia; 2 School of Health Sciences, Western Sydney University, Sydney, Australia; 3 Mental Health Services, South Western Sydney Local Health District, Camden and Campbelltown Hospital, Campbelltown, Australia; 4 AIS Performance, Australian Sports Commission, Canberra, Australia; 5 Discipline of Clinical Psychology, Graduate School of Health, University of Technology Sydney, Sydney, Australia

**Keywords:** Disordered eating, Eating disorder, Body image, Athlete, Sport

## Abstract

**Background:**

Despite the high prevalence of disordered eating and eating disorders amongst elite athletes, it remains unclear whether risk factors and psychological processes align with those in the general population or if there are unique sport-factors associated with heightened risk. This cross-sectional study investigated if sport-specific factors (including pressures and psychological processes) explained additional variance in elite athletes’ disordered eating symptoms, controlling for established population-general risk factors. Current elite athletes (*N* = 178, 72.4% female, mean age = 23.9, standard deviation age = 7.0) completed online surveys assessing disordered eating, body dissatisfaction, perfectionistic traits, population-general and sport-specific pressures, as well as general (thin-ideal, muscular-ideal) and athlete-specific (drive for leanness for performance, athletic identity) psychological processes.

**Results:**

Disordered eating was highly prevalent, with 78.2% of athletes reporting at least moderate risk, 46.4% at least high risk, and 20.6% very high risk. Controlling for demographic covariates and population-general pressures, sport-specific *pressures* explained significant additional variance (13.5%) in disordered eating. Even when controlling for perfectionistic traits, greater weight pressures in sport (*β* = .35) was uniquely associated with greater disordered eating. In a separate multivariate analysis controlling for covariates and general psychological processes, athlete-specific *psychological* *processes* explained significant additional variance (15.5%) in disordered eating. Even when controlling for body dissatisfaction, greater drive for leanness for performance (*β* = .17) and athletic identity (*β* = .13) were uniquely associated with greater disordered eating.

**Conclusions:**

These findings support evidence that elite athletes may experience dual pressures and psychological processes associated with disordered eating: those congruent with appearance-oriented models and others independent of appearance. This duality should be considered in the modification of interventions for disordered eating in elite athletes.

**Key points:**

1. Disordered eating was highly prevalent in a sample of 178 elite adult athletes, with 78.2% reporting at least moderate risk for having related symptoms or behaviours.
2. Greater *weight* *pressures* *in* *sport* was significantly associated with greater disordered eating, even when controlling for demographic covariates and population-general appearance-related pressures from family and the media.
3. Both appearance-based (drive for thinness, body dissatisfaction) and non-appearance (drive for leanness for performance, athletic identity) psychological processes were uniquely associated with greater disordered eating.

**Supplementary Information:**

The online version contains supplementary material available at 10.1186/s40798-024-00791-9.

## Background

Despite elevated risk for disordered eating amongst elite athletes [1, 2] and the associated deleterious implications for athlete health and performance [1, 3], empirical support remains unclear regarding correlates in this unique population. In the only athlete-specific model of disordered eating proposed to date, Petrie and Greenleaf [4] hypothesised that athletes experienced pressures both within their sporting environment (sport-specific pressures) and as part of general life (population-general pressures) regarding their eating, exercise, and bodies which could lead to disordered eating. Further, based on well-established models of disordered eating in the general population (e.g., tripartite influence model [5], dual-pathway model [6]), Petrie and Greenleaf [4] proposed that these relationships were mediated through psychological processes, namely internalisation of appearance ideals and subsequently body dissatisfaction. Athletes who compete at an elite level (e.g., national, international, professional, National Collegiate Athletic Association Division 1 [NCAA D1]; [7]) and who are immersed within the sporting environment may experience greater exposure to these “pressures” and “psychological processes”, making elite athletic status a moderating factor that increases their risk of disordered eating. Clearly, central to this model is the consideration of if and how elite athletes experience *unique* precipitating and maintaining factors for disordered eating compared with the general population [8].

Regarding the sport-specific versus population-general *pressures* in the model, findings indicate that both may be related to disordered eating in athletes. For example, greater perceived societal appearance-pressures (e.g., from the media) was associated with greater disordered eating in cross-sectional findings of adolescent Brazilian male athletes (72% elite [9]) and cross-sectional and longitudinal findings of United Kingdom adult male and female elite and non-elite athletes [10, 11]. In contrast, mere exposure to the sporting environment, measured through years in sport and hours spent training, was not associated with disordered eating [9–11]. Rather, athletes’ subjective experiences of pressures within their sporting environment to lose weight or be thin (e.g., from coaches, teammates, spectators) has been concurrently linked with greater body dissatisfaction and disordered eating symptoms in cross-sectional studies of NCAA D1 female gymnasts and swimmers/divers [12] and male NCAA collegiate athletes [13], and prospectively in longitudinal studies of NCAA D1 female gymnasts and swimmers/divers [14]. Further, athletes within certain sporting environments may experience greater pressure than others. Some sports have an inherent focus on thinness and weight-loss such as those which include judgements based on aesthetic (e.g., gymnastics), rules around meeting certain weight targets (e.g., combat sports), explicit anti-gravitational components (e.g., rock climbing, high jump), or those which promote leanness for endurance (e.g., long-distance running). These sport categories (aesthetic, weight-class, anti-gravitational, endurance) have previously been classed together as *lean* sports [15] with consistent findings of small effects indicating higher prevalence of disordered eating for these athletes compared with those competing in sports which do not have this inherent focus on leanness (i.e., non-lean sports), such as ball sports (e.g., basketball), technical sports (e.g., sailing), power sports (e.g., sprinting), and non-lean winter sports (e.g., snowboard cross) [2, 7, 16, 17]. Thus, in line with Petrie and Greenleaf [4], there is correlational and emerging prospective evidence that disordered eating in athletes may be associated with subjective pressures from both within and outside of the sporting environment.

Other potentially important sport-specific pressures have received relatively limited empirical investigation to date. A recent systematic review and meta-synthesis of 38 qualitative studies into elite athletes’ experiences with disordered eating [18] identified specific pressures to be associated with disordered eating, such as greater perceived power imbalance between coaches and athletes (e.g., athletes feeling unable to express their views), more frequent body monitoring procedures (e.g., weighing, skin-fold tests, body composition scans), rigid views of a single “ideal body” within a sport, and discrepancies between sports and population-general body ideals (e.g., a sports body ideal for muscularity and strength but a population-general ideal of thinness without being too ‘bulky’). No quantitative investigations have yet examined whether these sport-specific pressures are associated with disordered eating in elite athletes, particularly when controlling for population-general pressures. Clarification of these relationships, i.e., how general and sport-specific pressures are *uniquely* associated with disordered eating, is a critical step in developing more targeted and effective interventions in this population.

The separation of broader societal versus sporting specific factors in Petrie and Greenleaf [4]’s model focused on the *pressures* experienced by athletes. These dual pressures were then postulated to lead to disordered eating through the core psychological processes of internalisation of appearance ideals and body dissatisfaction—a well-established pathway to disordered eating evidenced in the broader population [6]. However, this potentially overlooks the possibility of athlete-specific psychological processes that may act alongside (or interact with) these broader psychological processes to increase risk for disordered eating. For instance, in Fatt, George [18]’s systematic meta-synthesis, athletes reported that certain beliefs can maintain disordered eating, including overly identifying with being an athlete and judging one’s value based on performance, alongside beliefs that greater leanness will lead to improvements in one’s performance. To date, quantitative studies investigating these processes are rare but affirm their relevance for disordered eating. Voelker, Gould [19] found that a stronger sense of athletic identity was significantly associated with higher disordered eating scores in female figure skaters (31% elite). Similarly, Krentz and Warschburger [20] found that greater drive for leanness to improve performance was associated with greater disordered eating symptoms in adolescent elite athletes (64% female). Further, this drive for leanness for performance cross-sectionally mediated the positive association between pressures for thinness in the sporting environment and disordered eating, whilst sports-related body dissatisfaction did not [20]. When assessed longitudinally in adolescent elite athletes (59% female), greater drive for leanness for performance (but not sports-related body dissatisfaction) predicted greater disordered eating 12 months later, rather than the inverse direction [21]. As such, there is evidence that in addition to the dual pathways of population-general and sporting *pressures* outlined by Petrie and Greenleaf [4], there may also be dual pathways of general (e.g., appearance-ideal internalisation) and athlete-specific *psychological* *processes* which precipitate and maintain disordered eating in elite athletes. The extent to which these dual processes are uniquely and independently associated with disordered eating remains unknown, and investigation of these relationships will further inform interventions.

Relatedly, it is unclear whether these athlete-specific psychological processes are associated with disordered eating *independent* of body dissatisfaction. Whilst Petrie and Greenleaf [4]’s model indicated that pressures and psychological processes, whether general or sport-specific, lead to disordered eating *through* body dissatisfaction, the empirical evidence for this is unclear. Several cross-sectional findings report positive associations between body dissatisfaction and disordered eating in athletes [7, 22]. However, several other findings suggest that athletes who reported higher disordered eating than non-athlete controls also report *equivalent* or even *lower* body dissatisfaction versus these same non-athlete controls [16, 23, 24]. This is supported by qualitative reports of athletes engaging in disordered eating behaviours for several non-appearance-related reasons, including attempts to meet sport-specific rules and regulations (e.g., meeting weight requirements for competition in certain sports, or ‘making weight’ [25, 26]) or the aforementioned drive for leanness for performance [27, 28]. If disordered eating in elite athletes is associated with athlete-specific psychological processes *independent* of body dissatisfaction, current interventions which primarily address body dissatisfaction as the central mechanism of change [29, 30] may need to be modified. Comparing general and athlete-specific psychological processes within a multivariate model, controlling for body dissatisatisfaction, will help clarify which processes to target to improve interventions for elite athletes.

Finally, it is important that models adjust for the influence of known demographic and personality correlates. Several demographic variables are suggested to be related to disordered eating [31], including being female, being younger, and having a lower body mass index (BMI). Notable amongst personality correlates, certain perfectionistic personality traits have been proposed as risk factors for disordered eating in athletes [32] and non-athletes alike [33].

This study thus aimed to examine and contrast the potency of general versus sport-specific correlates of disordered eating amongst elite athletes, including (1) population-general versus sport-specific pressures; and (2) population-general versus athlete-specific psychological processes. It was hypothesised that both general and sport-specific pressures would explain a unique amount of variance in athlete’s disordered eating, and these findings would hold when controlling for demographic covariates and perfectionistic personality traits. Further, it was hypothesised that athlete-specific psychological processes would explain a unique amount of variance in athlete’s disordered eating, and these findings would hold when controlling for demographic covariates, internalisation of appearance ideals (e.g., drive for thinness, drive for muscularity), and body dissatisfaction.

## Method

### Participants and Procedures

Participants were from the first wave of the ASPIRE (Addressing Sporting Pressures on athletes’ body Image and Relationships with Eating and exercise) study—a 3-wave longitudinal survey investigating body image and disordered eating in elite athletes. Current and former athletes, who had competed at the national, international, professional, or NCAA DI level, completed the baseline online survey between March-September 2023. Participants had the option to receive a $20 Australian dollars ($15 United States dollars) gift card or go in the draw to win a $100 Australian dollars gift card for their participation in the baseline survey. This flexible approach to participant compensation was used to incentivise participation.

Several recruitment strategies were used, including social media advertising, through elite sporting bodies (including National Institute Networks [NINs], and National Sporting Organisations [NSOs]), and snowball sampling. Social media advertising included “posts” and “stories” promoting the study, with a link for further information about the study (the study’s website and/or the Qualtrics link). This included posts from the study’s account with tags for the partnered NINs and NSOs, paid social media advertisements through Meta (reaching 86 349 accounts and 649 link clicks), and promotion by “paid” advertisers (e.g., athletes or clinicians promoting the study on their social media accounts and receiving a $50 Australian dollar token of thanks).

All potential participants were directed to a Qualtrics link, which included information about the study, a consent form, and screening questions on athlete status, competition level and age. Whilst the online advertising aided recruitment, several spammers (i.e., non-legitimate responders) completed the baseline survey. Thus, although 2960 respondents consented to the baseline survey, systematic steps were undertaken to identify likely spammers (see Supplementary 1) leaving 238 elite athletes (179 current, 59 former) who were deemed legitimate responders. An additional participant was excluded from analysis due to failing at least 2 of the 3 attention checks, leaving a final sample of 178 current elite athletes competing across various sports (mean[*M*]_Age_ = 23.9, standard deviation [SD]_Age_ = 7.0; 72.4% female, 27.0% male, 0.6% other gender; see Table 1 for full summary of demographic data). The unequal distribution across demographic variables was due to the self-selected method of participant recruitment.

**Table 1:** Descriptive statistics for the numeric and categorical variables

Numeric variables	*M* (*SD*)
Age	23.9 (7.0)
BMI	24.7 (4.8)
Hours training/competing per week	18.5 (6.9)
Disordered eating (ADE)	33.0 (12.3)
Eating disorder symptoms (EDE-QS)	8.5 (7.3)
Clinical impairment (CIA)	10.4 (10.1)
Body dissatisfaction (Mi-BoD)	53.0 (24.5)
Family pressures (SATAQ-4R)	1.8 (1.1)
Media pressures (SATAQ-4R)	2.8 (1.3)
Body monitoring frequency	2.7 (1.8)
Perceived pressure for a single “ideal” body in sport*	5.7 (2.6)
Body image duality*	4.9 (2.0)
Weight pressures in sport (WPS)	2.5 (1.0)
Perfectionism (Concern Over Mistakes)	25.2 (8.7)
Power imbalance (ELQ-PD reversed)	2.3 (0.8)
Drive for muscularity (SATAQ-4R)	3.8 (0.9)
Drive for thinness (SATAQ-4R)	2.9 (1.2)
Drive for leanness for performance (ATHLETE)	11.4 (4.5)
Athletic identity (ATHLETE)	21.4 (5.6)

Categorical variables	*N* (%)
*Gender*	
Males	47 (27.0)
Females	126 (72.4)
Other	1 (0.6)
*Lean* *vs.* *non-lean* *sport-type*	
Lean	77 (44.3)
Non-lean	88 (50.6)
Unclear	9 (5.2)
*Country* *of* *birth*	
Australia	138 (79.3)
Other	36 (20.7)
Self-identification as Aboriginal and/or Torres Strait Islander	3 (1.7)
Self-identification as a para athlete	14 (8.1)
*Disordered* *eating* *risk* *(ADE)*	
Minimal risk (0–24)	37.00 (21.8)
Moderate risk (25–32)	54 (31.8)
High risk (33–44)	44 (25.9)
Very high risk (45+)	35 (20.6)
EDE-QS above cut-off score (15+)	36 (21.4)
CIA above cut-off score (16+)	40 (24.4)

*M* mean, *SD* standard deviation; *BMI* body mass index; *ADE* athletic disordered eating scale; *EDE-QS* eating disorder examination-questionnaire short-form; *CIA* clinical impairment assessment; *Mi-BoD* multifaceted instrument for body image disturbance; *SATAQ-4R* sociocultural attitudes towards appearance questionnaire-4-revised; *WPS* weight pressures in sport; *ELQ-PD* empowering leadership questionnaire-participative decision-making.

*These constructs were measured via summed scores on two 5-point Likert scales (1 = *Strongly* *disagree* to 5 = *Strongly* *agree*).

### Measures

*Demographic* *and* *sporting* *information* Participants answered questions on age, gender (self-reported gender identity), country of birth, self-identification of Aboriginal and/or Torres Strait Islander ethnicity, average hours spent training and competing each week in sport, self-identification of being a para athlete, and sport(s) type. Sport(s) type was used to categorise athletes as either competing in at least one ‘lean’ sport (i.e., endurance, aesthetic, weight-class, or anti-gravitational sports), only ‘non-lean’ sports (i.e., ball, technical, power, and non-lean winter sports), or unclear (e.g., if an athlete only stated ‘athletics’)—in accordance with Martinsen and Sundgot-Borgen [15].

*Disordered* *eating* Disordered eating was assessed using the Athletic Disordered Eating (ADE) screening tool [34]. The ADE was developed and validated in male and female adult athletes to assess risk for a broad spectrum of disordered eating behaviours and symptoms. Participants respond to 17 items on a 5-point Likert scale: 0 = *Never,* 1 = *Rarely,* 2 = *Sometimes,* 3 = *Often,* 4 = *Always*. Total scores were summed creating a possible range of 0–68, with higher scores indicating greater risk of disordered eating. Buckley, Lassemillante [34] validated the ADE against the cut-off score for a clinical eating disorder on the Eating Attitudes Test—26 [35] identifying four categories of risk for disordered eating: minimal risk (total ADE < 25), moderate risk (ADE = 25–32) with 99.3% sensitivity and 27.3% specificity, high risk (ADE = 33–44) with 96.8% sensitivity and 49.7% specificity, and very high risk (ADE > 44) with 79.9% sensitivity and 84.7% specificity. Internal consistency was adequate in the current sample (McDonald’s *Ω* = 0.90). Other established screeners of eating disorder symptoms in the general population were also included: the Eating Disorder Examination—Questionnaire Short-form (EDE-QS) and the Clinical Impairment Assessment (CIA). The EDE-QS [36] has an established cut-off score of ≥ 15 indicating a likely eating disorder in the adult general population [37]. The CIA has an established cut-off score of 16 + indicating a likely eating disorder in the adult general population [38]. Although these measures have not been validated specifically in an athlete population, their internal consistency in the current sample was good (McDonald’s *Ω* = 0.90 and 0.95, respectively).

*Population-general* *(sociocultural)* *pressures* *and* *psychological* *processes* Two subscales from the Sociocultural Attitudes Towards Appearance Questionnaire-4-Revised (SATAQ-4R) were used to assess perceived appearance-based pressures from (1) family and (2) the media. The pressures from ‘peers’ subscale was not included for parsimony and due to likely overlap between athletes’ social and sporting networks. Two additional subscales from the SATAQ-4R were used to assess the internalisation of appearance ideals, namely drive for thinness and drive for muscularity. Separate versions of the SATAQ-4R have been validated for adult males versus adult females in the general population. Athletes responded via a 5-point Likert scale (1 = *Definitely* *Disagree* to 5 = *Definitely* *Agree*) to each item, which were averaged for total subscale scores. Higher average scores for each subscale indicated greater perceived pressure/drive for the appearance ideal. Although these measures have not been specifically validated in an athlete sample, internal consistency was adequate in the present sample for males and females respectively (McDonald’s *Ω*: pressures from family = 0.92 and 0.93, pressures from the media = 0.95 and 0.92, drive for thinness = 0.75 [note that because this subscale only has two items for males, Cronbach’s *α* was used rather than McDonald’s *Ω*] and 0.88, drive for muscularity = 0.88 and 0.88).

#### Sport-Specific Pressures

*Weight* *pressures* *in* *sport* Perceived pressures within the sporting environment for appearance or weight-loss were assessed using the Weight Pressures in Sport (WPS) scale, including separate versions for males (WPS-M [39], 12 items) and females (WPS-F [40], 11 items). Athletes responded on a 6-point Likert Scale (1 = *Never* to 6 = *Always*), with higher averaged scores indicating greater pressures for appearance or weight-loss. These scales have been previously validated in young adult elite collegiate athletes [39, 40] and internal consistency was adequate in the current sample (McDonald’s *Ω* = 0.86 and 0.86).

*Perceived* *power* *imbalance* *between* *coach* *and* *athlete* A modified version of the ‘Participative decision-making’ subscale of the Empowering Leadership Questionnaire (ELQ-PD [41]) was used to assess athletes’ perceptions of power balances with their coach/training staff. Athletes responded to six items on a 5-point Likert Scale (1 = *Strongly* *disagree* to 5 = *Strongly* *agree*), with average scores reversed so that higher scores indicated greater perceived power imbalance. Items were modified by replacing “work group members” with “athletes”, and “team leaders” with “coach/training staff” (see Supplementary 2). The internal consistency of the modified ELQ-PD was adequate in the current sample (McDonald’s *Ω* = 0.93).

*Body* *monitoring* *within* *sport* We devised a question asking athletes if any of the following monitoring procedures were part of their training or competition for their sport, including *weighing,* *skinfold* *testing,* *energy* *intake,* *dual-energy* *X-ray* *absorptiometry* *(DXA)* *scans,* *other*, or *none* *of* *the* *above*. Athletes who selected *none* *of* *the* *above* were coded as 0. The others were then asked, *“How* *often* *are* *these* *monitoring* *tools* *used* *as* *part* *of* *your* *sports* *training* *and/or* *competition,* *excluding* *any* *additional* *checking* *you* *may* *do”* and response options included: 1 = *Less* *than* *once* *per* *month*, 2 = *Every* *month*, 3 = *Every* *few* *weeks*, 4 = *About* *once* *per* *week*, 5 = *More* *than* *once* *per* *week*.

*Pressure* *for* *a* *single* *‘ideal’* *body* *in* *sport* Athletes rated how strongly they felt pressure within their sport to obtain a single ‘ideal’ body (1) shape and (2) weight, using a 5-point Likert scale (1 = *Strongly* *disagree* to 5 = *Strongly* *agree*). Scores were summed (Cronbach’s *α* = 0.89), with a higher score indicating greater perceived pressure for a single ‘ideal’ body in sport.

*Body* *image* *duality* Athletes responded to two items using a 5-point Likert scale (1 = *Strongly* *disagree* to 5 = *Strongly* *agree*): *“the* *"ideal"* *body* *shape* *for* *my* *sport* *is* *similar* *to* *the* *"ideal"* *body* *shape* *in* *broader* *society”* and *“the* *"ideal"* *body* *weight* *for* *my* *sport* *is* *similar* *to* *the* *"ideal"* *body* *weight* *in* *broader* *society”*. Higher summed scores (Cronbach’s *α* = 0.86) indicated greater perceived alignment in body ideals between their sport and broader society.

#### Athlete-specific psychological processes

Two subscales from the ATHLETE Questionnaire [42] were used to measure (1) drive for leanness for performance, and (2) athletic identity. Athletes responded on 5-point Likert scales (1 = *Strongly* *disagree* to 5 = *Strong* *agree*) for the 4-item short-form drive for leanness for performance subscale [20] and the 6-item athlete identity subscale. Total scores were summed, with higher scores indicating greater drive for leanness for performance and athletic identity, respectively. These subscales have been validated in elite young adult male and female athletes [20, 42] and demonstrated adequate internal consistency on the present study (McDonald’s *Ω* = 0.81 and 0.81, respectively).

#### Perfectionism

The Concern over Mistakes subscale from the Multidimensional Perfectionism Scale [43] was used to measure a perfectionistic trait which has consistently been associated with greater eating disorder psychopathology [33]. Athletes responded to the nine items on a 5-point Likert scale (1 = *Strongly* *disagree* to 5 = *Strong* *agree*). Total scores were summed (McDonald’s *Ω* = 0.91), with higher scores indicating greater trait perfectionism.

#### Body dissatisfaction

Body dissatisfaction was assessed using the Multifaceted Instrument for Body Image Disturbance (MI-BoD [44]). The MI-BoD was developed and validated in young adult undergraduate students, community adolescents, and a clinical sample to assess body image disturbances across six domains: dissatisfaction, overvaluation, preoccupation, fear of weight gain, body checking, and body exposure [44]. Athletes responded to 20 items using a 6-point Likert scale (1 = *Never* to 6 = *Always*), with summed scores indicating greater body dissatisfaction. The items were originally developed to avoid biases towards certain body-ideals (e.g., only the thin ideal) or genders (e.g., only females) and so was considered appropriate for the present sample of male and female elite athletes. Athletes’ body dissatisfaction could be measured without biased assumptions about their perceived body “ideals” (example items include: “I hated my body shape” or “I felt dissatisfied with my body shape or size”). The MI-BoD scale has not been validated specifically in an athlete population, but internal consistency in the current sample was adequate (McDonald’s *Ω* = 0.98).

### Analysis Plan

The order of the presentation questions in the survey was: demographic and sporting information, measures of disordered eating and body dissatisfaction (ADE, EDE-QS, MI-BoD, CIA), body monitoring, SATAQ-4R, WPS, ATHLETE (drive for leanness for performance, athletic identity), pressures for a single ‘ideal’ body in sport, body image duality, perceived power imbalance, perfectionism, height and weight (to calculate BMI). Bivariate analyses were conducted using independent *t*-tests for categorical correlates and Pearson’s correlations for numeric correlates. When assumptions of normality appeared to be violated (through Shaprio-Wilk tests and inspection of histograms), equivalent non-parametric analyses were conducted (i.e., Mann–Whitney *U* tests and Spearman’s rank correlation coefficients). Effect sizes were calculated and interpreted in line with existing guidelines [45], for example Cohen’s *d* for *t-*tests (0.2, 0.5, 0.8 for small, medium, and large effects, respectively) and *r* correlation coefficients (0.1, 0.3, 0.5 for small, medium, and large effects, respectively). Hierarchical multivariate linear regression analyses were used to investigate which correlates were significantly and uniquely associated with disordered eating, controlling for the other variables. The first model investigated hypothesis 1, first whether sport-specific pressures was uniquely associated with disordered eating beyond the variance explained by population-general pressures, and second when controlling for perfectionism. Gender and BMI were included as potential covariates in step 1, followed by population-general pressures in step 2, sport-specific pressures (if significant bivariate correlates of disordered eating) in step 3, and perfectionism in step 4. The second model investigated hypothesis 2, whether athlete-specific psychological processes was uniquely associated with disordered eating, first beyond the variance explained by drive for appearance ideals, and second when controlling for body dissatisfaction. Gender and BMI were included as potential covariates in step 1, followed by drive for thinness and muscularity in step 2, athlete-specific psychological factors (if significant bivariate correlates of disordered eating) in step 3, and body dissatisfaction in step 4. All assumptions were met for these two models, and no issues of multicollinearity were detected in either model (all tolerance at or above 0.52 and 0.37, respectively). Significance levels were adjusted to account for a paper-wide 5% false discovery rate using Benjamini and Hochberg (1995) procedure. The first raw *p* value to exceed the Benjamini–Hochberg-adjusted *p*-value corresponding to a false discovery rate of 5% was *p* = 0.023.

## Results

The proportion of missing data for each variable ranged between 2.2 and 13.5% (mean = 7.0%). Missing variables were handled via pairwise deletion.

### Descriptive Statistics

Descriptive statistics for the 178 included current elite athletes are presented in Table 1. Approximately 1 in 5 athletes scored in the *very* *high* *risk* range for disordered eating on the ADE. This proportion was comparable to the proportion of athletes who scored above the cut-off scores for a clinical eating disorder on the EDE-QS (21.4%) and the CIA (24.4%) (with moderate agreement; Kappa = 0.53), providing confidence in the validity of the ADE, the main outcome measure used in this study. An additional 3 in 5 athletes scored in the *moderate*–*high* risk range for disordered eating on the ADE, with only 1 in 5 athletes scoring in the *minimal* risk range.

### Bivariate Statistics

Bivariate correlations with disordered eating are presented in Table 2. Higher disordered eating on the ADE was significantly associated with greater appearance-based pressures from the media, weight pressures in sport (large effects), greater appearance-based pressures from family, pressure for a single ideal in sport, and concern over mistakes (medium effects). Of the psychological processes, higher disordered eating was significantly associated with higher drive for muscularity (small effect) and thinness (large effect), drive for leanness for performance (large effect), and athletic identity (small effect). Female athletes (*M* = 35.0, *SD* = 12.5) scored significantly higher on the ADE than male athletes (*M* = 27.8, *SD* = 10.4), *t*(167) = 3.48, *p* < 0.001, *d* = 0.63 (medium effect); however, there was no significant difference in ADE scores comparing athletes competing in lean (*M* = 31.2, *SD* = 11.4) vs. non-lean sports (*M* = 34.9, *SD* = 12.5), *t*(160) = 1.96, *p* = 0.052, *d* = 0.31 (small effect).

**Table 2:** Bivariate correlations with disordered eating (ADE)

	ADE	2	3	4	5	6	7	8	9	10	11
Correlation coefficients (*p* values) between ADE scores and demographic variables, population-general and sport-specific pressures											
2. BMI	.19^+^ (.026)										
3. Age	.08^+^ (.349)	.06 (.445)									
4. Family pressures (SATAQ-4R)	**.35**^**+**^ **(<** **.001)**	**.26** **(.001)**	− .12 (.129)								
5. Media pressures (SATAQ-4R)	**.46**^**+**^ **(<** **.001)**	.01 (.859)	− .01 (.903)	**.38** **(<** **.001)**							
6. Hours training/competing	− .03 (.736)	− .02 (.807)	− .13 (.100)	.04 (.584)	− .05 (.544)						
7. Body monitoring frequency	.10^+^ (.214)	.01 (.889)	.03 (.726)	.02 (.801)	− .08 (.283)	− .07 (.374)					
8. Perceived pressure for a single “ideal” body in sport*	**.43** **(<** **.001)**	− .02 (.839)	− .09 (.251)	**.21** **(.007)**	**.45** **(<** **.001)**	.11 (.155)	.12 (.143)				
9. Body image duality*	− .06^+^ (.485)	.05 (.570)	.07 (.426)	.08 (.360)	.14 (.095)	− **.15** **(.066)**	− .10 (.222)	.16 (.045)			
10. Weight Pressures in Sport (WPS)	**.49**^**+**^ **(<** **.001)**	.13 (.107)	.07 (.350)	**.37** **(<** **.001)**	**.43** **(<** **.001)**	.08 (.335)	**.21** **(.006)**	**.57** **(<** **.001)**	.08 (.316)		
11. Perfectionism (COM)	**.45** **(<** **.001)**	.09 (.251)	− .12 (.118)	**.22** **(.006)**	**.38** **(<** **.001)**	.03 (.713)	.04 (.605)	**.38** **(<** **.001)**	.05 (.518)	**.34** **(<** **.001)**	
12. Power imbalance (ELQ-PD reversed)	.14^+^ (.106)	.13 (.110)	.04 (.624)	.18 (.024)	.09 (.260)	**.23** **(.003)**	.03 (.756)	**.22** **(.007)**	− **.21** **(.012)**	**.30** **(<** **.001)**	**.21** **(.010)**

	ADE	13	14	15							
*Correlation* *coefficients* *(p* *values)* *between* *ADE* *scores* *and* *general* *and* *athlete-specific* *psychological* *processes*											
13. Drive for muscularity (SATAQ-4R)	**.24**^**+**^ **(.003)**										
14. Drive for thinness (SATAQ-4R)	**.62** **(<** **.001)**	**.18** **(.018)**									
15. Drive for leanness for performance (ATHLETE)	**.61**^**+**^ **(<** **.001)**	.17 (.036)	**.51** **(<** **.001)**								
16. Athletic Identity (ATHLETE)	**.26**^**+**^ **(.002)**	**.23** **(.003)**	.18 (.023)	.12 (.133)							

*ADE* athletic disordered eating scale; *BMI* body mass index; *SATAQ-4R* sociocultural attitudes towards appearance questionnaire-4-revised; *WPS* weight pressures in sport scale; *COM* concern over mistakes; *ELQ-PD* empowering leadership questionnaire–participative decision-making.

Bolded values indicates significance at the *p* < .023 level; ^+^Indicates Spearman’s correlation coefficient.

*These constructs were measured via summed scores on two 5-point Likert scales (1 = *Strongly* *disagree* to 5 = *Strongly* *agree*)

### Multivariate Model Examining Pressures

As seen in Table 3, after controlling for gender, BMI, and general population pressures (appearance-related pressure from family and from the media), adding sport pressures in step 3 explained a significant amount of additional variance in ADE scores (13.5%). Congruent with hypothesis 1, in the penultimate model, greater athlete disordered eating was significantly and uniquely associated with greater weight pressures in sport. In the final model, when adding perfectionism (concern over mistakes), greater disordered eating remained significantly associated with greater weight pressures in sport, whilst also being significantly associated with concern over mistakes.

**Table 3:** Multivariate regression analyses at each step for disordered eating (ADE): sociocultural and sport-specific pressures

	Step 1				Step 2				Step 3				Step 4			
	Δ*F*(2, 150) = 6.42 Adj *R*^*2*^ = .07, *p* = .002				Δ*F*(2, 148) = 20.29 Adj *R*^*2*^ = .26, *p* < .001				Δ*F*(3, 145) = 11.11 Adj *R*^*2*^ = .38, *p* < .001				Δ*F*(1, 144) = 8 .26 Adj *R*^*2*^ = .41, *p* = .005			
	LCI	UCI	Std. Error	Std. Beta	LCI	UCI	Std. Error	Std. Beta	LCI	UCI	Std. Error	Std. Beta	LCI	UCI	Std. Error	Std. Beta
Gender	**2.33**	**10.95**	**2.18**	**0.24**	− 3.74	5.03	2.22	0.02	− 1.90	6.28	2.07	0.08	− 2.27	5.74	2.03	0.06
BMI	− 0.01	0.79	0.20	0.15	− 0.14	0.60	0.19	0.09	− 0.17	0.51	0.17	0.07	− 0.20	0.47	0.17	0.05
Family pressures (SATAQ-4R)					**0.61**	**4.13**	**0.89**	**0.21**	− 0.30	3.01	0.84	0.12	− 0.30	2.93	0.82	0.12
Media pressures (SATAQ-4R)					**2.09**	**5.38**	**0.83**	**0.38**	0.21	3.55	0.85	0.19	− 0.19	3.12	0.84	0.15
Perceived pressure for a single “ideal” body in sport*									− 0.35	1.25	0.40	0.09	− 0.56	1.03	0.40	0.05
Weight pressures in sport (WPS)									**2.32**	**6.62**	**1.09**	**0.36**	**2.23**	**6.42**	**1.06**	**0.35**
Power imbalance (ELQ-PD reversed)									− 1.92	2.12	1.04	0.03	− 1.92	2.12	1.02	0.01
Perfectionism (COM)													**0.09**	**0.49**	**0.10**	**0.20**

Bolded values indicates significance at the *p* < .023 level.

*ADE* athletic disordered eating scale; *LCI* lower 95% confidence interval; *UCI* upper 95% confidence interval; *BMI* body mass index; *SATAQ-4R* sociocultural attitudes towards appearance questionnaire-4-revised; *WPS* weight pressures in sport scale; *COM* concern over mistakes; *ELQ* empowering leadership questionnaire–participative decision-making.

*Measured via summed scores on two 5-point Likert scales (1 = *Strongly* *disagree* to 5 = *Strongly* *agree*)

### Multivariate Model Examining Psychological Processes

As can be seen in Table 4, after controlling for gender, BMI, and general psychological processes (drive for thinness and muscularity), adding athlete-specific psychological processes (drive for leanness for performance and athletic identity) in step 3 explained a significant amount of additional variance in ADE scores (15.5%). Congruent with hypothesis 2, in the penultimate model, after controlling for the other variables, greater disordered eating was significantly associated with greater drive for thinness, drive for leanness for performance, and athletic identity. In the final model, when adding body dissatisfaction, only drive for leanness for performance, athletic identity, body dissatisfaction, and female gender were significantly associated with disordered eating.

**Table 4:** Multivariate regression analyses at each step for disordered eating (ADE): psychological processes

	Step 1				Step 2				Step 3				Step 4			
	Δ*F*(2, 150) = 6.42 Adj *R*^*2*^ = .07, *p* = .002				Δ*F*(2, 148) = 42.04 Adj *R*^*2*^ = .40, *p* < .001				Δ*F*(2, 146) = 26.26 Adj *R*^*2*^ = .55, *p* < .001				Δ*F*(1, 144) = 8.26 Adj *R*^*2*^ = .41, *p* = .005			
	LCI	UCI	Std. Error	Std. Beta	LCI	UCI	Std. Error	Std. Beta	LCI	UCI	Std. Error	Std. Beta	LCI	UCI	Std. Error	Std. Beta
Gender	**2.33**	**10.95**	**2.18**	**0.24**	− 7.16	0.95	2.05	-0.11	− 5.90	1.11	1.77	− 0.09	− **6.26**	− **0.62**	**1.43**	− **0.12**
BMI	− 0.01	0.79	0.20	0.15	0.01	0.66	0.17	0.13	− 0.14	0.45	0.15	0.06	− 0.19	0.27	0.12	0.01
Drive for Muscularity (SATAQ-4R)					0.00	3.72	0.94	0.13	− 0.60	2.69	0.83	0.07	− 1.09	1.51	0.66	0.01
Drive for thinness (SATAQ-4R)					**5.08**	**8.08**	**0.76**	**0.64**	**2.55**	**5.50**	**0.75**	**0.39**	− 0.60	2.07	0.68	0.07
Drive for leanness for performance (ATHLETE)									**0.80**	**1.52**	**0.18**	**0.42**	**0.16**	**0.79**	**0.16**	**0.17**
Athletic identity (ATHLETE)									**0.13**	**0.62**	**0.12**	**0.17**	**0.08**	**0.47**	**0.10**	**0.13**
Body dissatisfaction (MI-BoD)													**0.27**	**0.41**	**0.04**	**0.68**

Bolded values indicates significance at the *p* < .023 level.

*ADE* athletic disordered eating scale; *LCI* lower 95% confidence interval; *UCI* upper 95% confidence interval; *BMI* body mass index; *SATAQ-4R* sociocultural attitudes towards appearance questionnaire-4-revised; *MI-BoD* multifaceted instrument for body image disturbance

## Discussion

This quantitative study examined cross-sectional correlates (population-general vs sport-specific) of disordered eating in current elite athletes. Disordered eating was highly prevalent across athletes: one fifth of the athletes scored in the *very* *high* *risk* range for disordered eating, with a similar proportion scoring above the clinical cut-off scores on the EDE-QS and the CIA. As hypothesised, sport-specific pressures explained a significant and unique amount of variance in athletes’ disordered eating scores, controlling for demographic correlates, population-general pressures, and perfectionistic personality traits. However, contrary to our hypothesis, population-general pressures were not significant correlates when controlling for sport-specific pressures. The second hypothesis was also supported, with athlete-specific psychological processes explaining a unique amount of variance in athletes’ disordered eating scores when controlling for general psychological processes and body dissatisfaction. These findings provide further support for a dual processes (general and sport-specific) model of disordered eating in elite athletes [4].

Several population-general and sport-specific pressures were significantly associated with greater disordered eating in bivariate analyses; however, when included together in the multivariate model only *weight* *pressures* *in* *sport* remained significantly associated. This relationship was robust and remained significant even when controlling for perfectionism, a replicated strong correlate of disordered eating [33]. Contrastingly, other pressures were not significantly associated with disordered eating in the multivariate model (family pressures, media pressures, pressure for a single “ideal” body in sport) or in bivariate analyses (hours training/competing, frequency of body monitoring procedures, body image duality, power imbalance). These quantitative findings build on previous qualitative findings [18] and are congruent with previous cross-sectional and longitudinal findings that specific subjective pressures for weight and thinness within sport are associated with greater disordered eating, whilst other aspects of the sporting environment (e.g., mere exposure through time training/competing) may be less relevant [9–12, 14]. Further, our results support previous findings that greater perceived societal appearance-pressures (e.g., from the media) are associated with higher risk for disordered eating across various athlete populations [9–11]; however, extending on these findings we found that these relationships were no longer significant when controlling for sport pressures and perfectionistic personality traits. As such, whilst elite athletes may experience both population-general and sport-specific pressures, the pressures from within the sporting environment may be more relevant for disordered eating. These findings support and extend Petrie and Greenleaf [4]’s model of disordered eating in athletes, which proposes dual pathways but does not delineate the relative strength of each pathway.

Support for hypothesis 2 highlights the independent roles of both population-general and athlete-specific *psychological* *processes* for disordered eating. Each of the psychological processes (drive for thinness, drive for muscularity, drive for leanness for performance, athletic identity) were associated with greater disordered eating. This is congruent with previous findings that general psychological processes [11] and athlete-specific psychological processes [19–21] were associated with greater disordered eating risk in athletes when considered individually. However, the present study extended these findings by investigating the *unique* relationships of these psychological processes through multivariate analyses, finding that drive for thinness, drive for leanness for performance, and athletic identity were each uniquely associated with greater disordered eating, whilst drive for muscularity was no longer significantly associated. Thus, both appearance and non-appearance-driven psychological processes may be associated with greater disordered eating in elite athletes and these processes are, at least in part, independent.

Extending this further, when controlling for appearance-based concerns by including body dissatisfaction in the model, the two athlete-specific psychological processes (drive for leanness for performance, athletic identity) remained significant predictors of disordered eating, whilst drive for thinness did not. This aligns with previous research, including a study where drive for leanness for performance correlated with greater disordered eating while controlling for body dissatisfaction in German adolescent elite athletes competing in aesthetic sports [20]. Thus, whilst internalisation of appearance ideals and body dissatisfaction may be associated with disordered eating in athletes per Petrie and Greenleaf [4], our findings extend the model to include other psychological processes (i.e., drive for leanness for performance, athletic identity) which are athlete-specific and non-appearance-driven as possible additional, and possibly overlapping, pathways for disordered eating in elite athletes.

These athlete-specific psychological processes may be theoretically aligned with Fairburn, Cooper [30]’s cognitive behavioural model of eating disorders, which purports the overvaluation of weight and shape as central to eating disorder aetiology. However, for athletes, the cognitions underlying this overvaluation may be varied, including appearance-related reasons (as per the general population) but also performance-related reasons. Specifically, athletes who overly identify with “being an athlete” and who strongly believe that their performance will improve if they become leaner may place inordinate value on losing weight, leading to disordered eating symptoms. Our findings suggest that these appearance and non-appearance reasons may overlap but may also be independent in explaining disordered eating in elite athletes. Although our findings are cross-sectional and do not allow for imputation of causation, previous longitudinal analyses have indicated directionality from greater drive for leanness for performance at baseline predicting increased disordered eating over time, and not the inverse [21]. Thus, there is strong rationale for future longitudinal studies investigating dual population-general and athlete-specific aetiological pathways for disordered eating in elite athletes, including both pressures and psychological processes.

### Implications

The broad variance of ADE scores in this study across the *moderate*, *high*, and *very* *high* risk ranges highlights how any athlete may be at risk for varying levels of disordered eating symptomology. This provides further support for a spectral view of disordered eating in elite athletes, rather than exclusively focusing on “eating disorders” per se [1]. Further, although female athletes scored higher than male athletes for disordered eating in the present study and in previous findings [46, 47], disordered eating was still prevalent amongst males, with the average ADE score in the *moderate* risk range. Additionally, whilst several findings have previously indicated small but significant effects of greater risk for athletes competing in lean versus non-lean sports [7, 16, 17], this was not replicated in the present study. This challenges notions of at-risk athletes ‘archetypes’ (e.g., female athletes participating in lean sports) and narrow views of eating disorder symptomology (e.g., low BMI, vomiting [48]), encouraging broader screening for disordered eating across entire athlete populations. Sporting organisations should develop and implement policies for managing athletes at *all* levels of risk, including primary, secondary, and tertiary interventions [49].

The findings of independent relationships between population-general and athlete-specific correlates with disordered eating provide directions for improving preventative and treatment interventions targeting disordered eating specifically in elite athletes. There is a clear responsibility for sporting organisations and communities to address pressures within their sporting environment to reduce disordered eating risk in their athletes, rather than attributing blame to greater societal pressures for body ideals or the athletes’ own characteristics (e.g., gender, personality types). Several proposals for changes in sporting culture, policy, and training methods have been documented elsewhere [1, 49, 50], with examples including reducing body exposure in sporting attire (which has been proposed as problematic in previous qualitative studies [18, 51]), policies around safe body monitoring and weighing procedures, and education for coaches, training staff, media and commentators, and athlete support networks.

Modifications of current prevention and treatment programs for disordered eating may also be warranted to address athlete-specific psychological processes. Current evidence-based interventions for the prevention (e.g., The Body Project [29]) and treatment of eating disorders (e.g., CBT-E [30]) in the general population typically target appearance concerns as a central mechanism of change. Direct translation of these interventions for elite athletes may only address part of the dual pressures and psychological processes, neglecting the potentially more key beliefs around performance and identity. This may explain, at least in part, the relatively limited effectiveness of prevention programs for eating disorders in athletes to date [52–55] compared with findings in the general population [56]. In developing future interventions for disordered eating in elite athletes, appearance *and* non-appearance (i.e., drive for leanness for performance and athletic identity) psychological processes should be considered. This may be through (1) an integrated approach addressing these pathways at once, (2) a sequential approach addressing each pathway consecutively, or (3) an assessment-driven approach which matches athletes with interventions based on their scores on measures for each psychological process. Such interventions are currently lacking, and the development and evaluation for each potential approach should be a priority for future research.

### Limitations

These findings should be interpreted with consideration of the following limitations. First, the cross-sectional design cautions against making assumptions of causality in these relationships. For example, experiencing greater pressures for thinness in the sporting environment may lead to greater disordered eating; however, athletes with greater disordered eating concerns may also have attentional and memory biases towards comments about weight and food. This limitation is planned to be addressed, at least in part, through the follow-up surveys at 6 months and 12 months after baseline as part of the broader ASPIRE study, allowing for longitudinal analyses to be conducted. Second, athletes were a self-selected sample (i.e., opt-in), likely leading to a bias in our sample towards participants who were interested in “body image, eating, and exercise”. This bias relates primarily to the data on the rates of disordered eating in our sample, and we warn readers against interpreting this as prevalence data. Investigation of the ADE scores did not indicate a restricted range of disordered eating in our sample, suggesting a low likelihood of this bias impacting on our specific hypotheses. Third, the sample primarily included Australian female athletes. However, the congruence of these findings with previous research in male and female athletes from across the world [12, 20] gives tentative confidence to the study’s generalisability. Fourth, over 2000 suspected illegitimate spam responses were removed from the study. Whilst we undertook several steps to distinguish between legitimate and spam responses (see Supplementary 1), it is possible that some spam responses were included or that legitimate responses were excluded from the final analyses. Finally, although the SATAQ-4R and the WPS have different items for males versus females we treated these scales uniformly across gender. Separate analyses for male versus female athletes would have been insufficiently powered and we instead opted to control for gender in each of the multivariate models.

## Conclusions

Any elite athlete may be at risk for disordered eating, regardless of gender, age, body composition, or sport-type. Broad screening is needed to detect those at risk. Both population-general and athlete-specific factors have independent associations with disordered eating, including pressures (pressures for thinness and weight-loss from within sport) and psychological processes (drive for thinness, drive for leanness for performance, and athletic identity). These correlates can be considered in practice through changes to policy, procedure, and interventions for disordered eating, and in future research through longitudinal investigations of the direction of these relationships and the potential mediating role of the psychological processes.

## Supplementary Information

Additional file1.

Additional file2.

## Data Availability

The datasets generated and/or analysed during the current study are not publicly available due to the participant’s consent but are available from the corresponding author on reasonable request.

## Abbreviations

ADE: Athletic disordered eating scale
BMI: Body mass index
CBT-E: Cognitive behavioural therapy for eating disorders
CIA: Clinical impairment assessment
COM: Concern over mistakes
DXA: Dual-energy X-ray absorptiometry
EDE-QS: Eating disorder examination-questionnaire short-form
ELQ-PD: Empowering leadership questionnaire-participative decision-making
*M*: Mean
Mi-BoD: Multifaceted instrument for body image disturbance
NCAA: National collegiate athletics association
NINs: National institute networks
NSOs: National sporting organisations
NSW: New South Wales
SATAQ-4R: Sociocultural attitudes towards appearance questionnaire-4-revised
*SD*: Standard deviation
WPS: Weight pressures in sport
