# Supplementary material for: Comparing Population-General and Sport-Specific Correlates of Disordered Eating Amongst Elite Athletes: A Cross-Sectional Study
Source: Sports Med Open. 2024 Nov 12;10:123. doi: 10.1186/s40798-024-00791-9 (PMC11557852; doi:10.1186/s40798-024-00791-9)
Supplement: Supplementary file 1 — Additional file1. [file 40798_2024_791_MOESM1_ESM.pdf]

Journal: Sports Medicine – Open

Title: Comparing Population-general and Sport-specific Correlates of Disordered Eating Amongst Elite Athletes: A Cross-sectional Study

Authors: Scott J. Fatt[1], Emma George[1, 2], Phillipa Hay[1, 3], Nikki Jeacocke[4], & Deborah Mitchison[1, 5]

[1] Translational Health Research Institute, School of Medicine, Western Sydney University, Sydney, Australia

[2] School of Health Sciences, Western Sydney University, Sydney, Australia

[3] Mental Health Services, South Western Sydney Local Health District, Camden and Campbelltown Hospital, Campbelltown, Australia

[4] AIS Performance, Australian Sports Commission, Canberra, Australia

[5] Discipline of Clinical Psychology, Graduate School of Health, University of Technology Sydney, Sydney, Australia

Corresponding author: Scott J. Fatt

s.fatt@westernsydney.edu.au

Supplementary 1. Steps for identifying likely spammers.

1) Responses from before the use of paid social media advertisements were considered *legitimate* responses (*n* = 91).
2) All responses received after the paid social media advertisements which included the $20 reimbursement for participating (*n* = 2869) were considered as potential spam.
3) Of these, 176 respondents did not pass the screening questions and so were excluded. An additional 146 responses were deemed *legitimate* through triangulation of the timing of recruitment, the respondent's reported sport organisation, their reported sport, and their name (e.g., an athlete self-reporting as a rower for the NSW Institute of Sport within 48 hours of when the advertisement was sent out to that population), leaving 2547 potential spammers.
4) Next, respondents were deemed as likely spammers for any of the following reasons (assessed sequentially).
   a. Any of the 3 attention checks incorrect (*n* = 1675)
   b. Having a duplicate IP address (*n* = 157)
   c. Completing the full survey in less than 9 minutes (*n* = 48)
   d. Reporting a sport that was not considered a professional or elite sport (e.g., "dog surfing", "keep aways"; *n* = 36)
5) This left 631 potential spammers. They were each sent an email explaining that spammers had signed up for the study and requesting for them to confirm their involvement in the study, providing two pieces of information: 1) their sport of competition; and 2) their age they started in sport.
6) We received email responses from only 48 respondents. Those who did not reply to the email were deemed likely spammers and excluded. For those who did reply to the email, each response was assessed based on triangulation of 1) whether the

participant’s responses were consistent with their responses in the survey; 2) appropriate responses to the email request (e.g., some email responses only said “Yes, I will participate” without answering the prompt questions); 3) searching the participant’s name online to determine their athlete status. Of these, only 1 participant was considered a *legitimate* responder and their data was included in the final analyses.
