# Supplementary material for: Comparing Population-General and Sport-Specific Correlates of Disordered Eating Amongst Elite Athletes: A Cross-Sectional Study
Source: Sports Med Open. 2024 Nov 12;10:123. doi: 10.1186/s40798-024-00791-9 (PMC11557852; doi:10.1186/s40798-024-00791-9)
Supplement: Supplementary file 2 — Additional file2. [file 40798_2024_791_MOESM2_ESM.pdf]

Journal: Sports Medicine – Open

Title: Comparing Population-general and Sport-specific Correlates of Disordered Eating Amongst Elite Athletes: A Cross-sectional Study

Authors: Scott J. Fatt[1], Emma George[1, 2], Phillipa Hay[1, 3], Nikki Jeacocke[4], & Deborah Mitchison[1, 5]

[1] Translational Health Research Institute, School of Medicine, Western Sydney University, Sydney, Australia

[2] School of Health Sciences, Western Sydney University, Sydney, Australia

[3] Mental Health Services, South Western Sydney Local Health District, Camden and Campbelltown Hospital, Campbelltown, Australia

[4] AIS Performance, Australian Sports Commission, Canberra, Australia

[5] Discipline of Clinical Psychology, Graduate School of Health, University of Technology Sydney, Sydney, Australia

Corresponding author: Scott J. Fatt

s.fatt@westernsydney.edu.au

Supplementary 2 – Modified version of the Empowering Leadership Questionnaire – Participative Decision-making subscale

Please answer how strongly you agree with each of the following statements.
My coaching/training staff…

1. Encourages athletes to express ideas/suggestions
2. Listens to athletes' ideas and suggestions
3. Uses athletes' suggestions to make decisions that affect them
4. Gives all team members a chance to voice their opinions
5. Considers ideas of athletes even when they disagree with them
6. Makes decisions that are based only on their own ideas
